# A Simple and Effectual Means of Administering Salvarsan

**Published:** 1912-07

**Authors:** 


### A Simple and Effectual Means of Administering Salvarsan.

Fowler (*Indian Medical Gazette*) says that finding the ordinary procedure that is recommended very tedious and troublesome, he tried the mixture of salvarsan with olive oil, and was surprised at the results. The local pain was practically nil, and the patients improved rapidly under the treatment, as is recorded by the injection of salvarsan in saline solution. The procedure adopted is as follows: A Roux's syringe with a short needle of fairly large bore is sterilized by boiling in water. An ounce of pure olive oil is boiled in a small aluminum basin, and the oil is allowed to cool, or if one is in a hurry the basin is put into some cold water.

Two cubic centimeters of oil is drawn up in the syringe with the needle on—the piston is drawn well up to the top and the oil is shaken up, so that the oil adheres to the sides of the glass barrel. The piston is then completely drawn out, a finger being placed on the point of the needle (which is lowermost), so that the oil may not run out; next the salvarsan is thrown into the barrel, and 4 c.c. of oil is poured on the salvarsan direct from the aluminum basin. The piston is then replaced, and as soon as the washer engages the barrel, the syringe is turned up, so that the needle is uppermost. The screw-cap which is on the piston rod is then pushed up and screwed home, and the syringe is violently agitated.

The salvarsan mixes with the oil and forms a uniform emulsion. This is injected intramuscularly into the gluteal region.

The points that should be noted are:

1. The piston of the syringe should not be of metal. The syringes supplied for salvarsan injections by chemists are unsuitable for this purpose as the acid salvarsan attacks the metal and the piston jams.

2. The needle must be of wide bore.

3. Care should be taken when the piston is being replaced, the glass barrel being held firmly in its metallic casing with the left hand. The author advises those wishing to try this method to practice it with some simple oil.

4. There should be no hurry in the mixing, as the whole of the salvarsan will mix up with the oil. He has had no caking of the salvarsan in the syringe.—*Charlotte Medical Journal.*

